# Exploring Differential Connexin Expression across Melanocytic Tumor Progression Involving the Tumor Microenvironment

**DOI:** 10.3390/cancers11020165

**Published:** 2019-02-01

**Authors:** Gergo Kiszner, Peter Balla, Barna Wichmann, Gabor Barna, Kornelia Baghy, Istvan Balazs Nemeth, Erika Varga, Istvan Furi, Bela Toth, Tibor Krenacs

**Affiliations:** 1 Department of Pathology and Experimental Cancer Research, Semmelweis University, H-1085 Budapest, Hungary; kiszner.gergo@gmail.com (G.K.); ballapeti81@gmail.com (P.B.); barna.gabor@med.semmelweis-univ.hu (G.B.); bcory6@gmail.com (K.B.); 2 2nd Department of Internal Medicine, Semmelweis University, H-1088 Budapest, Hungary; wwbarna@yahoo.com (B.W.); furiistvan88@gmail.com (I.F.); 3 Department of Dermatology and Allergology, University of Szeged, H-6720 Szeged, Hungary; estvannemeth@yahoo.com (I.B.N.); varga.erika@med.u-szeged.hu (E.V.); 4 Department of Dermatology, Venereology and Dermato-oncology, Semmelweis University, H-1085 Budapest, Hungary; toth.bela@med.semmelweis-univ.hu

**Keywords:** melanocytic tumor progression, malignant melanoma, connexin isotype expression, gap junctions, direct cell-cell communication, melanoma microenvironment

## Abstract

The incidence of malignant melanoma, one of the deadliest cancers, continues to increase. Here we tested connexin (Cx) expression in primary melanocytes, melanoma cell lines and in a common nevus, dysplastic nevus, and thin, thick, and metastatic melanoma tumor progression series involving the tumor microenvironment by utilizing in silico analysis, qRT-PCR, immunocyto-/histochemistry and dye transfer tests. Primary melanocytes expressed *GJA1*/Cx43, *GJA3*/Cx46 and low levels of *GJB2*/Cx26 and *GJC3*/Cx30.2 transcripts. In silico data revealed downregulation of *GJA1*/Cx43 and *GJB2*/Cx26 mRNA, in addition to upregulated *GJB1*/Cx32, during melanoma progression. In three melanoma cell lines, we also showed the loss of *GJA1*/Cx43 and the differential expression of *GJB1*/Cx32, *GJB2*/Cx26, *GJA3*/Cx46 and *GJC3*/Cx30.2. The dominantly paranuclear localization of connexin proteins explained the ~10–90 times less melanoma cell coupling compared to melanocytes. In melanocytic tumor tissues, we confirmed the loss of Cx43 protein, fall of cell membrane and elevated paranuclear Cx32 with moderately increased cytoplasmic Cx26 and paranuclear Cx30.2 positivity during tumor progression. Furthermore, we found Cx43, Cx26 and Cx30 proteins upregulated in the melanoma adjacent epidermis, and Cx43 in the tumor flanking vessels. Therefore, differential connexin expression is involved in melanocytic tumor progression where varying connexin isotypes and levels reflect tumor heterogeneity-related bidirectional adaptive interactions with the microenvironment.

## 1. Introduction

Despite easy access and visibility of the skin and organized screening programs, cutaneous melanoma is one of the most fatal cancers, which shows an increasing incidence in white-skinned populations worldwide [1]. Even thin melanomas may give rise to distant metastases associated with poor treatment response and prognosis [2,3]. Connexin (Cx) communication channels play essential roles in coordinating epidermal functions, emphasized by the link between mutations of five skin-expressed connexins—Cx26, Cx30, Cx30.3, Cx31, and Cx43—and several human hereditary skin syndromes [4]. So far, two of these isotypes, Cx43 and Cx26, were shown to be common between epidermal keratinocytes and adjoining melanocytes that allow direct metabolic interactions between these cell types [5]. In spite of experimental data on the potential role of Cxs in controlling melanoma development and metastatic invasion, studies on melanocytic tumor progression in clinical cases are still incomplete.

Cxs and their channels play key roles in maintaining multicellular homeostasis in most tissues, including the skin (as has been discussed by several papers related to this issue), with particular importance of regulating cell death, proliferation [6] and migration [7]. They can also interact directly with a wide range of intracellular regulatory molecules, including adhesion, proto-oncogene and oncosuppressor proteins (“gap junction proteome”), to provide channel independent functions [8]. Therefore, Cxs either forming cell membrane channels or localized in the cytoplasm can influence fundamental cell functions. In carcinogenesis, Cxs have long been considered as tumor suppressors [9], e.g., by inhibiting cell growth and/or promoting apoptosis [10]. However, they can also be involved in tumor invasion, e.g., by supporting epithelial-mesenchymal transition (EMT) and tumor cell diapedesis through the vascular wall [11,12]. Cx channels can also contribute to bystander cell damage induced either by chemo- and radiotherapy or ischemic cell injury followed by infarction (“spreading depression”) [13]. Therefore, Cxs play context-dependent roles in tumors, and their expression pattern, the produced isotypes and subcellular localization, can be changed during tumor development and progression [14].

Partly controversial data have been reported regarding Cxs in melanocytes and melanocytic tumors. Most published data relate to Cx43 showing either its absence or significantly reduced expression in melanomas [15,16,17,18], which contributes to the loss of intrinsic control on tumor growth, death and metastasis. Functional studies of a gap junction-deficient mouse melanoma (B16-BL6) model showed that forced expression of Cx43 restored direct cell–cell communication (GJIC), and significantly reduced tumor cell proliferation and anchorage-independent growth, where growth inhibition was also confirmed in vivo in chicken chorioallantois membrane [5]. Also, in a human melanoma (FMS) cell line, overexpression of Cx43 supported TNF-α mediated apoptosis in vitro and reduced the number and size of lung metastatic tumor foci in a mouse xenograft model [19].

Other Cx isotypes have also been detected, however, with unclarified functions. Cx26 was found in vestibular melanocytes and in the epidermal and dermal microenvironment (in endothelial and stromal cells) of cutaneous melanomas [15,16,20,21]. Cx26 (in addition to Cx43) was reduced more in amelanotic versus the less aggressive melanotic canine oral melanomas [22]. Increased Cx30 expression was observed in the epidermal microenvironment of melanomas [15,16], adjacent to healthy/normal epidermal keratinocytes expressing a wide range of Cxs (Cx30.3, Cx31, Cx31.1, Cx37 and Cx45 Cx30, Cx26, Cx43) [23]. Further proof for the involvement of Cxs in the regulation of pigmented skin cells came from zebrafish (*Danio rerio*) studies showing that targeted mutations of the *gja5b* gene (Cx41.8; the orthologue of mammalian GJA5/Cx40), determined skin pattern, i.e., the spatial distribution of melanophore and xantophore cells both forming this channel isotype [24,25].

Here we studied Cx expression both at mRNA and protein levels using human primary melanocytes, melanoma cell lines and a large cohort of human clinical tumors representing stages of melanocytic tumor development and progression. In silico analysis of Cx transcript levels in melanoma progression groups and the functional testing of direct cell–cell communication in primary melanocytes compared with melanoma cell lines were also performed.

## 2. Results

### 2.1. In Silico Connexin Gene Expression Analysis

Relevant data from GEO database and the probes for Cx genes in Affymetrix arrays are shown in Table 1 and Table 2. Significant comparative gene expression results, including in silico findings, are summarized in Table 3. In datasets using GSE3189 arrays, melanomas showed downregulated *GJA1* (Cx43; LogFC = −4.1; *p* < 0.001), *GJB3* (Cx31; LogFC = −3.4; *p* < 0.001), *GJB5* (Cx31.1; LogFC = −3.3; *p* < 0.001) and upregulated *GJB1* (Cx32; LogFC = +1.5; *p* < 0.001) gene expression compared to nevi [26].

In GSE7553 arrays, metastatic melanomas showed downregulated *GJA1* (Cx43; LogFC = −1.9; *p* < 0.001), *GJB2* (Cx26; LogFC = −4.6; *p* < 0.001), *GJB3* (Cx31; LogFC = −3.2; *p* < 0.001), *GJB5* (Cx31.1; LogFC = −2.5; *p* < 0.001) and *GJB6* (Cx30; LogFC = −7.1; *p* < 0.001) gene expression compared to primary (including in situ) melanomas [27]. *GJA3*/Cx46 was also detected at a low level without difference between groups.

In GSE8401 arrays, metastatic melanomas from xenograft models showed downregulated *GJA1* (Cx43; LogFC = −2.8; *p* < 0.001), *GJB3* (Cx31; LogFC = −1.8; *p* < 0.001) and *GJB5* (Cx31.1; LogFC = −1.1; *p* < 0.001) gene expression compared to primary melanomas [28].

### 2.2. Connexin Gene Expression in Cultured Primary Melanocytes and Melanoma Cell Lines

**Figure 1:**
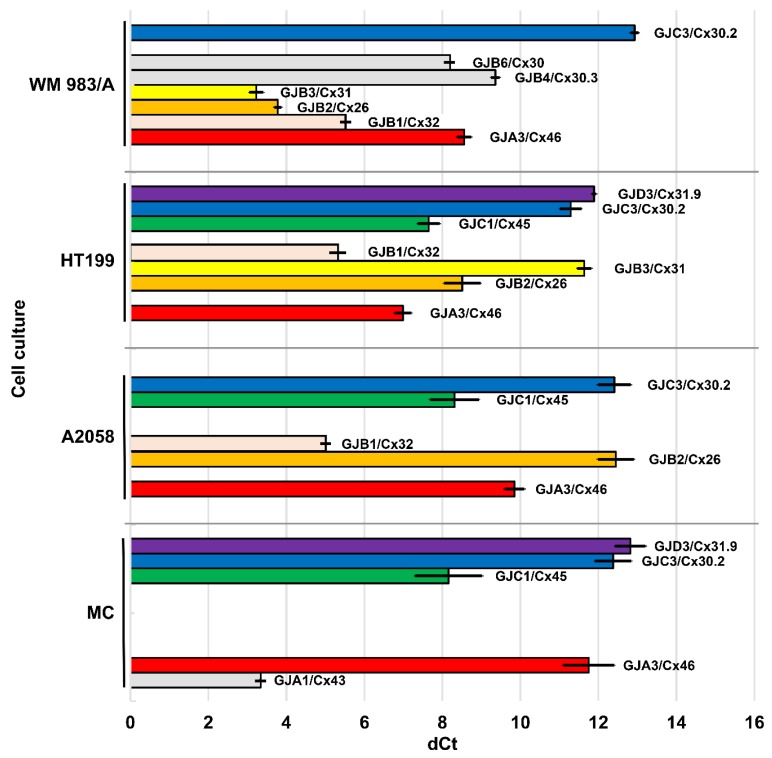
Connexin gene expression in cultured primary melanocytes (MC) and melanoma cell lines A2058, WM983/A and HT199. Threshold cycles of connexin gene expressions were compared to that of the housekeeping beta actin as a reference (dCt). Genes expressed in more than one cell line are colored. Black lines show ±SD of at least three independent isolations. Connexin genes expressed at highest levels (shortest bars) were *GJA1* (Cx43) in MC, as well as *GJB2* (Cx26) and *GJB3* (Cx31) in WM 983/A, and *GJB1*(Cx32) in all three melanoma cell lines.

Threshold cycles (CT) of GJ/Cx isotype expression were compared to those of the housekeeping beta actin (ACTB) gene as a reference (dCt) (Figure 1). While ACTB signal (mean Ct) was presented between the 17th and 23rd cycles, the expression of Cx genes was considered to be positive if its Ct was below 35.

*GJA1* (Cx43) gene was expressed only in primary melanocyte (MC) cultures (dCt = 3.34, SD = 0.03).

*GJA3* (Cx46) mRNA levels were lower in MC (dCt = 11.75; SD = 0.66) than in melanoma cell lines A2058 (dCt = 9.85; SD = 0.25), WM983/A (dCt = 8.56; SD = 0.18) and HT199 (dCt = 6.99; SD = 0.22). Compared with MC, relative quotient (RQ) was 16.544 in HT199, 8.595 in WM983/A and 3.741 in A2058. Significantly higher mRNA levels were detected in HT199 (*p* < 0.001), WM983/A (*p* < 0.001) and A2058 (*p* = 0.026) versus MC; in HT199 (*p* < 0.001) and WM983/A (*p* < 0.001) vs. A2058, and in HT199 vs. WM983/A (*p* = 0.028).

*GJB1* (Cx32) could not be detected in MC, but it was highly expressed in all three melanoma cell lines: WM983/A (dCt = 6.65; SD = 0.23), HT199 (dCt = 5.25; SD = 0.42) and A2058 (dCt = 4.92; SD = 0.14), though Hs.PT.56a.4848609 TaqMan probe could not detect *GJB1* mRNA in HT199 and A2058 cells.

*GJB2* (Cx26) was expressed in MC (dCt = 4.53; SD = 0.13) and in WM983/A (dCt = 3.78; SD = 0.09), but significantly lower expression in HT199 (dCt = 8.51; SD = 0.47; *p* < 0.001) and very low levels in A2058 (dCt = 12.45; SD = 0.46; *p* < 0.001). Compared with MC, RQ was 1.683 in WM983/A, 0.004 in A2058, and 0.063 in HT199. Differences between MC and WM983/A (*p* < 0.001), MC and A2058 (*p* < 0.001), MC and HT199 (*p* < 0.001) were significant.

*GJB3* (Cx31) showed high expression in WM983/A (dCt = 3.23; SD = 0.18), lower levels in HT199 (dCt = 11.64; SD = 0.18) but it was not detected in A2058.

*GJC1* (Cx45) showed significantly higher expression in HT199 (dCt = 7.65; SD = 0.28) than in MC (dCt = 8.16; SD = 0.86) (*p* < 0.001) and in A2058 (dCt = 8.31; SD = 0.63) and it was not detected in WM983/A. Compared to MC, RQ was 1.424 in HT199 and 0.899 in A2058.

*GJC3* (Cx30.2) was expressed at low levels in all samples: HT199 (dCt = 11.29; SD = 0.28), MC (dCt = 12.38; SD = 0.47), A2058 (dCt = 12.41; SD = 0.42) and WM983/A (dCt = 12.93; SD = 0.10). Compared to MC, RQ was 2.131 in HT199, 0.986 in A2058 and 0.687 in WM983/A. In HT199, *GJC3* expression was significantly higher than in A2058 (*p* = 0.033) or WM983/A (*p* = 0.031), and it was higher in MC (*p* = 0.024) or A2058 (*p* = 0.004) than in WM983/A.

*GJD3* (Cx31.9) mRNA was detected in HT199 (dCt = 11.89; SD = 0.05; RQ = 1.912) and less in MC (dCt = 12.82; SD = 0.39).

*GJB4* (Cx30.3) (dCt = 9.36; SD = 0.11) and *GJB6* (Cx30) (dCt = 8.20; SD = 0.03) gene expression was detected only in WM983/A cell line and no *GJA4* (Cx37), *GJB5* (Cx31.1), *GJB7* (Cx25), *GJC2* (Cx47), *GJD2* (Cx36) or *GJD4* (Cx40.1) mRNA expression was seen in any of the cell lines tested.

### 2.3. In Situ Detection of Connexin Protein Isotypes in Melanocytes and Melanocytic Tumors

**Figure 2:**
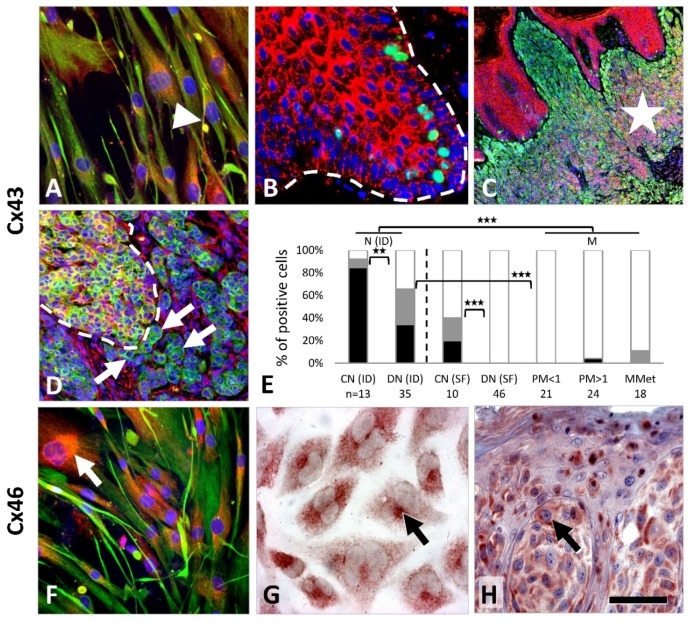
Cx43 (**A**–**E**) and Cx46 (**F**,**H**) protein expression (immunofluorescence: red; cell nuclei: blue) in melanocytes and melanocytic lesions. Cultured primary melanocytes show granular cytoplasmic and cell membrane (arrowhead) staining (**A**; vimentin intermediate filaments: green). Dense granular signal is seen in melanoma adjacent epidermal hyperplasia between keratinocytes and less surrounding all cells in the basal epidermal layer (along the broken line) including melanocytes (**B**; Ki67 positive proliferating keratinocytes: green). Strong reaction in the middle and basal regions in line with maturation in a common nevus (**C**; asterisk, melan **A**: green). A Cx43 positive nevus nest (within broken line) and adjacent stromal cells with Cx43 negative atypical tumor cells (arrows, melan **A**: green) in a nevus-based melanoma (**D**). Cx43 expression in common (CN) and dysplastic nevi (DN), in thin (PM < 1) and thick (PM > 1) primary as well as in metastatic (MMet) melanomas (*** *p* ≤ 0.001; ** *p* ≤ 0.01) (**E**). ID, intradermal; SF, superficial; black, >25%, gray, 5–25%, white, ≤5%. Cx46 immunoreactions show paranuclear (ER-Golgi) accumulation (arrows) in cultured melanocytes (**F**; vimentin: green), in HT199 metastatic melanoma cell line (**G**) and in a nodular melanoma (**H**). In immunoperoxidase reactions AEC (3-amino-9-ethylcarbazole, red) chromogen was used. Scale bar on **H** represents 40 μm on **A** and **F**; 50 μm on **B** and **H**; 150 μm on **C**, 80 μm on **D**, and 20 μm on **G**.

Cx43 (*GJA1*) immunostaining showed punctuate cytoplasmic and focal membranous pattern in primary melanocyte culture (MC), but no reaction in melanoma lines A2058, HT199 or WM983/A (Figure 2A). In tissue sections, Cx43 was detected as granular cytoplasmic and cell membrane reaction between epidermal keratinocytes and between them and melanocytes. All common nevi (13/13, 100%) and most dysplastic nevi (23/35, 66%) were positive but without a significant difference (Figure 2B–E). If weak reactions (≤25% of cells) were considered negative, the positivity rate was significantly higher in common nevi 11/13 (85%) than in dysplastic nevi 12/35 (34%) (*p* = 0.003). No or rare Cx43 reactions were seen in superficial regions of common nevi (1/10, 10%) and dysplastic nevi (0/46, 0%), as well as in primary (1/45, 2%) and metastatic melanomas (2/18, 11%).

Cx46 (*GJA3*) immunoreactions showed diffuse cytoplasmic and occasionally cell membrane localization in primary melanocytes and in WM983/A and A2058 cell lines, and dominantly paranuclear staining in HT199 melanoma cells (Figure 2F,G). In tissue samples, Cx46 reaction was paranuclear in all nevi (56/56, 100%) and in most melanomas (55/57, 96%), and rarely was cell membrane linked with no significant difference in frequency between these or any other tumor subgroups (Figure 2H).

**Figure 3:**
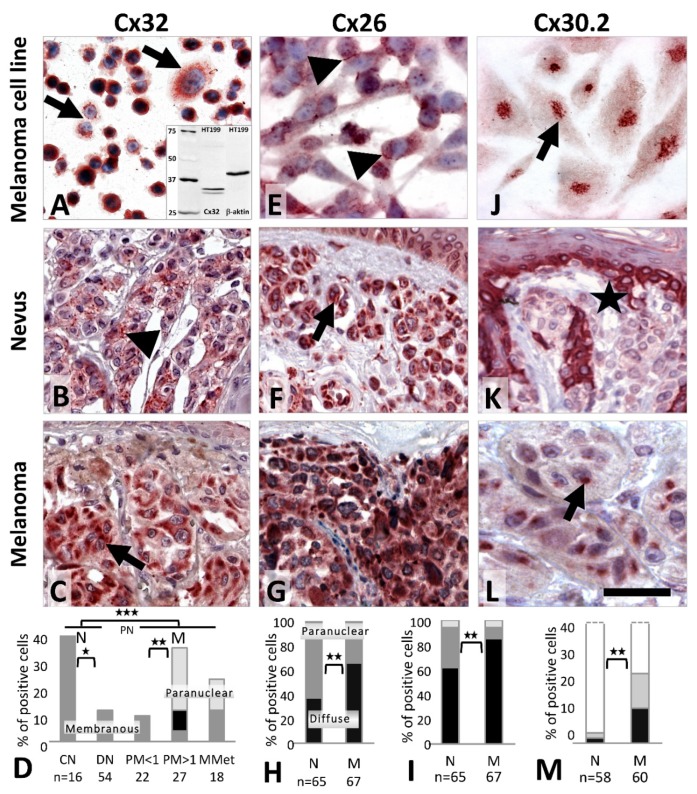
Cx32 (**A**–**C**), Cx26 (**E**–**G**) and Cx30.2 (**J**–**L**) immunoperoxidase reactions (AEC: red), and graphs summarizing Cx32 (**D**), Cx26 (**H**,**I**) and Cx30.2 (**M**) expression in melanocytic tumors. Cx32, (**D**) dark gray, membranous; light gray, paranuclear localization; or black, both; *** *p* ≤ 0.001; ** *p* = 0.002–0.010; * *p* = 0.011–0.05. Cx26, (**H**) black, diffuse; dark grey, paranuclear; (**I**) black, >75%; dark gray, 50–75%; light gray, 10–50%. (**M**) black, >25%; gray, 5–25%; white, <5%. Cx32 reactions show cytoplasmic localization in WM893/A melanoma cell line (**A**; inset: Western blot) and cell membrane positivity (arrowhead) in a dysplastic nevus (**B**), and dominantly paranuclear accumulation (arrow) in a thick melanoma (**C**). Cx26 reactions show cytoplasmic and cell membrane (arrowheads) localization in HT199 melanoma cell line (**E**), dominantly peri- and paranuclear (arrow) staining in a common nevus (**F**) and diffuse cytoplasmic staining in a metastatic melanoma (**G**), whose pattern is significantly different between these groups. Paranuclear Cx30.2 signals in HT199 melanoma cell line (**J**) and in a thick melanoma (**L)** but there is no reaction in a common nevus (**K**) where the tumor adjacent basal keratinocyte layer (asterisk, except melanocytes) is positive. Scale bar on **L** represents 40 μm on **A**; 50 μm on **B**, **C**, **F** and **G**; 30 μm on **E** and **L**, and 20 μm on **J**.

Cx32 (*GJB1*) protein was detected both in the vertical growth phase WM983/A (Figure 3A) and in the metastatic melanoma lines A2058 and HT199 as a granular and/or diffuse cytoplasmic reaction, while primary melanocytes were negative. In tissue sections, moderate cytoplasmic Cx32 signals were seen in 62/70 (89%) nevi and 65/67 (97%) melanomas. Membranous Cx32 localization was observed in 6/16 (38%) common nevi versus 6/54 (11%) dysplastic nevi (*p* = 0.023) (Figure 3B) and in 7/67 (10%) melanomas (including metastases). Paranuclear Cx32 staining was found in 8/27 (30%) thick melanomas (Figure 3C,D) and 2/18 (11%) metastases with no such reaction in thin melanomas (0/22, 0%; *p* = 0.006) or nevi (0/70, 0%) (*p* = 0.001).

Cx26 (*GJB2*) protein was detected as a cytoplasmic and occasionally cell membrane signal in melanocytes, and in A2058 and HT199 (Figure 3E) metastatic melanoma cells. In tissues, differential subcellular localization was observed. In nevi the reaction was dominantly paranuclear (Figure 3F) (41/65, 63%) compared to the diffuse cytoplasmic reaction in melanomas (Figure 3G,H) (44/67, 66%) (*p* = 0.002). Furthermore, there were significantly less Cx26 positive nevi (40/65, 62%) than melanomas (57/67, 85%) (*p* = 0.003) (Figure 3I).

In culture, Cx30.2 (*GJC3*) immunoreaction was seen in primary melanocytes and in the HT199 melanoma cell line (Figure 3J). In tissues, Cx30.2 was very rarely detected in common nevi (2/58, 3%) (Figure 3K) or dysplastic nevi (2/45, 4%) but it was more frequent in primary melanomas (14/60, 23%; 2/18, 11% of thin and 8/25, 32% of thick) and in metastatic cases (4/17, 24%) (Figure 3L,M) as a cytoplasmic and frequently paranuclear reaction.

### 2.4. Dye Transfer Analysis for Gap Junction Communication

**Figure 4:**
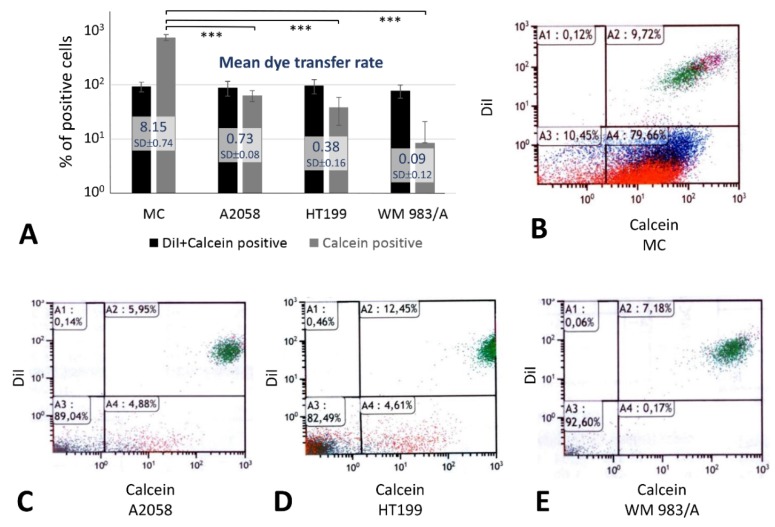
Functional dye-transfer tests using flow cytometry showing significantly higher rate of gap junctional direct transport of calcein from double labelled (Dil + calcein positive) cells (*** *p* < 0.001) into unlabeled touching cells in cultured primary melanocytes than in melanoma cell lines (**A**). Dye transfer rate (**A**) indicates the mean number of single calcein labelled cells coupled to each double labelled cell. Results show the mean ± standard deviation of three parallel experiments with each cell line. Representative examples of flow cytometry on melanocyte/MC (**B**), as well as A2058 (**C**), HT199 (**D**) and WM983/A (**E**) melanoma cultures.

Five hours after co-culturing 10^5^ Dil (1, 1′ dioctadecyl-3, 3, 3′, 3′-tetramethylindocarbocyanine perchlorate) and calcein (released in donor cells from its acetoxymethyl ester, calcein AM) double-positive cells with 9 × 10^5^ identical unlabeled cells, 79.66% of primary melanocytes, but only 4.88% of A2058, 4.61% of HT199 and 0.17% of WM 983/A melanoma cell lines, showed single calcein positivity indicating direct dye transfer though gap junctions among tumor cells (Figure 4). As a control, 10.45%, 89.04%, 82.49%, 92.6% of these cell types respectively were double negative and 9.72%, 5.95%, 12.45%, 7.18% of them respectively were Dil and calcein double positive.

### 2.5. Connexin Expression in Tumor Microenvironment

**Figure 5:**
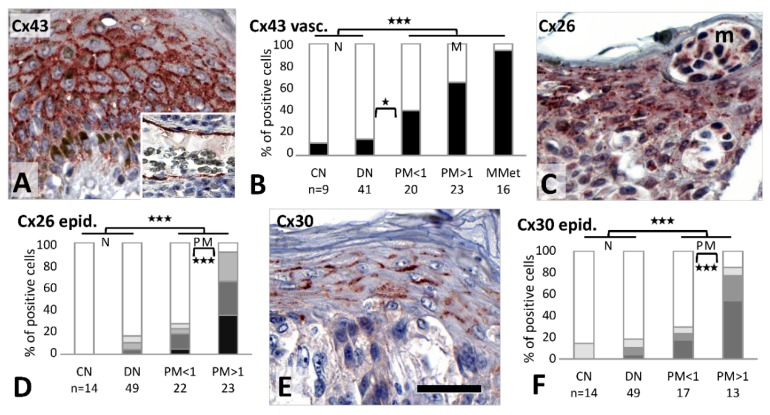
Connexin expression in melanocytic tumor microenvironment. Epidermal keratinocytes above a thick melanoma show Cx43 cell membrane reaction (red) in all layers besides melanocyte (brown) hyperplasia (**A**). Inset shows Cx43 positivity in lymphoid vascular endothelial cells of a metastatic melanoma. Summary of Cx43 expression in tumor adjacent vessels (**B**). Elevated granular Cx26 reaction in the epidermis surrounding melanoma nests (m) of a thick melanoma (**C**). Summary of tumor adjacent epidermal Cx26 expression (**D**). Cell membrane associated Cx30 reaction in suprabasal epidermal keratinocytes above a thin melanoma (**E**). Summary of tumor adjacent epidermal Cx30 expression (**F**). (**D** and **F**: white, no expression; light gray, only in stratum granulosum; medium gray, in uppermost layers; dark gray, in suprabasal layers; black, in all layers). Scale bar on **E** is 40 μm long and it represents 50 μm on **A** (including inset) and **B**. *** *p* ≤ 0.001.

In the tumor adjacent epidermis of primary melanomas, Cx43 immunoreaction was elevated and extended to all layers (Figure 5A), in contrast to the resting normal epidermis, where it is localized to the basal and suprabasal layers only. This upregulation was significantly more frequent than in nevi (19/43, 44% vs. 12/60, 20%; *p* = 0.01), and in thick than thin melanomas (14/22, 64% vs. 5/21, 24%, respectively; *p* = 0.014). Cx43 was also detected in dermal stromal cells of nevi and melanoma samples (see Figure 2B,D). Vascular and lymphatic endothelial cells were also significantly more frequently Cx43 positive in melanomas (38/59, 64%) than in nevi (7/50, 14%) (*p* < 0.001), and this showed a progressive tendency from common nevi (1/9, 11%), through dysplastic nevi (6/41, 15%), thin melanomas (8/20, 40%), thick melanomas (15/23, 65%) to metastases (15/16, 94%), with a significant increase from dysplastic nevi to thin melanomas (*p* = 0.049) (Figure 5B). Weak to moderate Cx46 positive cells in the dermal stroma, including vascular endothelial cells, were also seen around tumor nests, but without showing a major difference between groups. However, no stromal element in the dermis was Cx32 immunopositive.

Tumor adjacent epidermis also showed significantly higher Cx26 expression in melanomas (27/45; 60%) compared to nevi (8/63;13%) (*p* < 0.001), and this difference was also significant between thin (6/22, 27%) and thick (21/23, 91%) melanomas (*p* < 0.001) (Figure 5C,D).

Cx30 was also significantly more frequently expressed in epidermal keratinocytes surrounding primary melanomas (20/34, 59%) than nevi (11/63, 17%) (*p* < 0.001), involving more thick (15/17, 88%) than thin melanomas (5/17, 29%) (*p* = 0.001) (Figure 5E,F). Differential epidermal Cx expression of thick compared to thin melanomas showed 0.91 sensitivity and 0.77 specificity for Cx26, and 0.85 sensitivity and 0.71 specificity for Cx30.

Furthermore, strong Cx30.2 signal was seen in the tumor adjacent basal epidermal (stem cell rich) layer in all cases (see Figure 3K), which extended more to the superficial layers in 23/39 (59%) primary melanomas than in nevi (15/57; 26%) (*p* = 0.002).

## 3. Discussion

Malignant melanomas, caused by genetic predisposition and environmental factors such as UV irradiation, still shows an increasing incidence and poor survival rates in metastatic cases [1]. Depending on patient history, ~25% melanomas may arise from preexisting nevi, but most of those associated with chronic sun exposure evolve from in situ melanomas or dysplastic lesions [29]. Here we studied Cx expression during melanocytic tumor progression using in silico data analysis, primary melanocytes and melanoma cell lines, as well as a clinical melanocytic tissue series representing different stages of tumor evolution. Our results reveal diverse Cx isotype expression during melanocytic tumor progression including the loss of Cx43 protein with moderate recurrence in metastases and elevation of diffuse cytoplasmic Cx26. Both Cx30.2, a novel isotype detected also in melanocytes, and Cx32, which was missing from melanocytes, showed increasing frequency of paranuclear reaction through melanoma progression. Furthermore, Cx43, Cx26 and Cx30 proteins were upregulated in the melanoma adjacent epidermis, as was Cx43 in the tumor flanking lymphoid endothelia/vessels. Our data indicate the involvement of differential Cx expression in melanocytic tumor evolution where Cx alterations are likely related to the intra- and intergroup heterogeneity of tumor geno-/phenotypes and the bidirectional interactions with their microenvironment.

**Figure 6:**
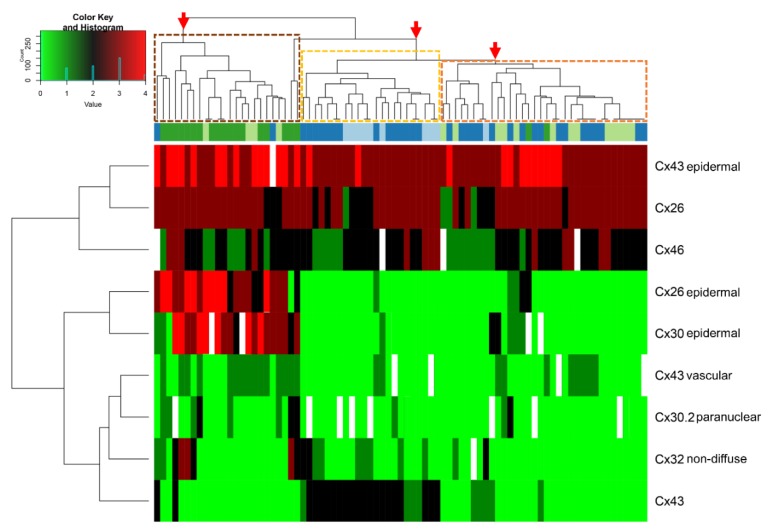
Unweighted hierarchical cluster analysis of connexin isotype expression in melanocytic tumor tissues and their microenvironments. Besides demonstrating tumor heterogeneity, three clusters (arrows) are separated from each other: (1) thick (dark green) and thin (pale green) melanomas (brown frame); (2) common (pale blue) and dysplastic nevi (dark blue; orange frame); and (3) a mixed group of thin melanomas and dysplastic nevi (light brown frame). Cases with missing data are in white.

Melanomas are made up of a high number of clones with diverse genetic (and epigenetic) dysregulation, which lead to exceptionally high intra- and intertumoral heterogeneity [30]. They also show a remarkable cellular plasticity involving reversible EMT and switching between differentiated and invasive phenotypes driven by both oncogenic signaling/genetic instability and environmental factors [31]. Varying levels of DNA methylation, histone post-translational modifications, chromatin remodeling and non-coding (micro- and long-) RNAs can further boost melanocytic tumor cell diversity [32]. The expression of several Cx isotypes during melanocytic tumor progression and their differing ranges even within the same group, as we observed, reflect this heterogeneity, particularly after malignant transformation. Diverse Cx expression also highlights the selective adaptation of tumor cells to microenvironmental (stromal, endothelial, epithelial, leukocytic) cues depending on their makeup [33]. The short half-lives of Cxs allow their rapid, transient and differential production [34] where the frequency of positive cells and subcellular localization of Cxs may occasionally differ among progression groups. Hierarchical clustering of our major Cx expression data in primary melanocytic tumor tissues and their microenvironments visually demonstrates this heterogeneity and diversity (Figure 6).

However, clustering into three major groups of partly distinct features can also be observed. The first group is dominated by thick melanomas characterized by lost Cx43 protein, elevated paranuclear Cx30.2 and Cx32 positive cases, in addition to increased epidermal Cx43, Cx26 and Cx30, and vascular Cx43 levels. The second includes common and dysplastic nevi featured by Cx43 expression both in the tumors and the surrounding epidermis with missing epidermal Cx26 and Cx30 proteins. The third group is a mixture of dysplastic nevi and thin melanomas mostly with Cx43 negativity both in the tumors and their microenvironments with practically no expression of Cx30.2. This pattern suggests a functional continuum between some dysplastic nevi and thin melanomas in line with the clinical observations showing that dysplastic nevi are risk factors of melanoma development [35]. Our observation that Cx43 expression showed by far the most significant reduction from benign to malignant lesions of intratumoral Cxs is in agreement with functional studies proving the privileged role of Cx43 (mainly forming gap junctions) as the chief tumor suppressor isotype [5,19]. We show further indirect proof for this by the concentration of cell membrane linked Cx43 protein in the central and vertical regions of nevi, in line with their known maturation (see Figure 2C) [36]. Cx43 gap junctions may also contribute to preventing Cx43 positive dysplastic nevi from malignant transformation, which could be verified in prognostic follow-up studies. Major reduction of Cx43 expression (to 2%) in line with malignant transformation and its cytoplasmic re-expression at metastatic sites (11%) suggests their dual role and involvement either in tumor suppression or tumor adaptation. This is supported by our dye transfer studies where the vertical growth phase melanoma cell line (WM793B) showed nearly 30-times less coupling than the metastatic ones (HT199 and A2058). These data are partly in agreement with a study showing that primary melanomas are Cx43 negative while their metastases reproduce this protein, which, however, cannot form gap junctions [37].

Nevertheless, intracytoplasmic Cx43 protein can still influence cell functions through interacting with dozens of intracytoplasmic regulatory molecules (“gap junction proteome”) [8]. Upregulation of Cx43 in melanoma-associated vascular endothelia, as we observed, supports the chance for Cx43 to be involved in tumor intra- and extravasation during metastatic tumor dispersion, as has been found by others in melanoma, breast and prostate cancers [38,39,40]. Cx30.2 protein, which we detected for the first time in melanocytes and their lesions (its gene probes were missing from the expression arrays [26,27,28]), was significantly more frequent in malignant than in benign lesions, but this involved only few positive cases. Nevertheless, the rare, strongly Cx30.2 positive melanomas might represent a clinically relevant subtype that requires further studies.

Tumor heterogeneity was also reflected by the differential Cx expression among the three invasive, metastatic melanoma cell lines, which altogether involved seven detectable isotypes (*GJA1*/Cx43, *GJB1*/Cx32, *GJB2*/Cx26, *GJA3*/Cx46, *GJB3*/Cx31, *GJC1*/Cx45, *GJD3*/Cx30.2) at mRNA and four (no Cx43 and unreliable Cx31 and Cx45), at protein level. These data are supported by other studies revealing mRNAs of Cx32, Cx31 and Cx45 in WM793B and 1205Lu human melanoma cells lines [41], as well as those of Cx43, Cx31, Cx31.1, Cx32 [26], Cx26 and Cx30 [27], and Cx46 [28], in human melanoma cases. In melanocytic tumor tissues we confirmed the expression of all the same Cx isotypes as in cell lines except Cx31 and Cx45, where controversial Western blot results did not allow validation of our antibodies for immunocyto-/histochemistry. On the other hand, we detected Cx32 mRNA and protein together for the first time in melanoma cell lines. Interestingly, one of the probes bridging through exon1 to exon3 could not detect *GJB1* mRNA in HT199 and A2058 metastatic cells, while using the other two probes targeting exon2 revealed strong expression in all three cell lines. The reason for negativity might be related to alternative splicing of GJB1 transcript in the metastatic cell lines, but not in the vertical growth phase (WM983/A) cells. Though other groups also found Cx32 transcripts in some melanomas [26,41], ours is the first, by using three different TaqMan probes and a Western blot-verified antibody, to show unequivocal evidence of both *GJB1* transcript and Cx32 protein in melanoma cell lines; and Cx32 protein in melanoma tissues.

Of important note, however, is that expression array data must be critically interpreted since most are not based on laser microdissection isolated pure tumor cell fractions [42]. Therefore, these samples may be contaminated by Cx transcripts of epidermal keratinocytes (i.e., *GJA1*/Cx43, *GJB2*/Cx26, *GJB3*/Cx31, *GJB5*/Cx31.1, or *GJB6*/Cx30) or of the stromal components [43]. This emphasizes the importance of our results gained in vitro in pure cell lines, and in situ both in cells and tissues, where our Western blot pretested antibodies allowed us determining the subcellular localization of Cxs in individual cells with a potential functional relevance.

In primary melanocytes we detected Cx43 and Cx46, and less Cx26 and Cx30.2, both at mRNA and protein level. However, only Cx43 protein showed cell membrane association, which further confirmed its exclusive role in forming gap junctions and being the major substrate of the 10–90 times less dye coupling when it was lost in melanoma cell lines (see Figure 4) despite their cytoplasmic Cx46, Cx32, Cx26 or Cx30.2 positivity. The frequent, exclusively paranuclear localization of these Cxs both in melanoma cell lines and tissues is consistent with their sticking in the endoplasmic reticulum trans-Golgi region without proper maturation [34]. In contrast, the diffuse cytoplasmic Cxs can potentially be involved in protein interactions and/or limited membrane transport and channel formation. However, we have tested gap junction coupling only, thus, functioning hemichannels or Cx-protein interactions [8,44] may also be in action during melanocytic tumor progression.

Melanoma cells escape direct proliferation control of keratinocytes by deregulating direct cell-cell interactions mediated by calcium-dependent cell adhesion (E-cadherin, P-cadherin, desmoglein) and Cxs [45]. Induction of Cx26 and Cx30 in melanoma adjacent keratinocytes reflects the impact of melanomas on their microenvironment probably through soluble factors [33]. This is in line with earlier reports and might have differential diagnostic importance since this feature is missing in benign nevi [16]. Cx26 and Cx30 upregulation was found to be positively correlated with keratinocyte proliferation [15], which could be driven by transforming growth factor-α (TGF-α) produced by melanomas [33]. Stimulation of Cx26 and Cx30 expression was also described at early skin wounding, where replenishment of the epidermis also requires keratinocyte proliferation [23,46].

Cx43 gap junctions are known to inhibit cell proliferation, this is why they are downregulated during early skin wounding where keratinocyte proliferation is essential [46]. On the contrary, upregulated Cx43 in chronic wounds delays healing and its specific inhibition with Cx mimetic peptides or antisense oligonucleotides are in phase III trial of potentiating diabetic wound healing [47]. Since most of our thick melanomas were exulcerated, the increased expression of Cx43 in the epidermis surrounding them is likely be related to chronic skin wounding [48].

Though Cx43 and Cx26 detected in melanocytes are also among those five isotypes (Cx30, Cx26, Cx43, Cx30.3 and/or Cx31) which mutations are linked to congenital skin lesions (and hearing loss syndromes), melanocyte dysfunctions have not been reported yet in these cases, which suggests that they are not affected [4,5].

The differing Cx transcript and protein levels we saw in the melanoma cell lines are possibly related to epigenetic regulation, e.g., by microRNAs and/or posttranslational protein modifications [32]. Some discordant or incoherent results among the available tissue studies on Cxs and melanomas may result from using diverse patient cohorts and/or antibodies of differing specificities and sensitivities, particularly when applied in formalin-fixed, paraffin-embedded tissues [49].

Further investigations should focus on correlations between Cxs and deregulated genotypes, diverse prognosis or therapy response of melanomas. Separate groups of different pathogenesis should include tumors based on genetic susceptibility, such as melanocortin 1 receptor (MC1R) polymorphism, and/or BRAFv600e, the GTPase NRAS, phosphatase and tensin homolog (PTEN), cyclin-dependent kinase inhibitor 2A (CDKN2A or p16^INK4^), or cyclin-dependent kinase 4 (CDK4) mutations [29].

## 4. Materials and Methods

### 4.1. In Silico Analysis of Connexin Isotype Expression in Melanocytes and Melanocytic Lesions

Data on connexin gene isotype expression in biopsies of human melanocytic tumors were obtained from publications uploaded to and freely accessible in the Gene Expression Omnibus (GEO) database (Table 2 and Table 3). Affymetrix Human Genome U133 Plus 2.0 or U133A gene chips were used in the collected data series. Expression data were tested after normalization using the robust GCRMA gene chip algorithm and the mean expression values were statistically analyzed.

### 4.2. Melanoma Cell Lines and Melanocyte Cultures

Human invasive melanoma cell lines A2058 (metastasis from brain) [50], HT199 (metastatic to the liver and lung) [51], and WM983/A (vertical growth phase) [52,53], were available in the tumor bank of the 1st Department of Pathology and Experimental Cancer Research, Semmelweis University, Budapest. They were grown in RPMI 1640 medium (PAN-Biotech GmbH, Aidenbach, Germany) supplemented with 5% fetal bovine serum (FBS) and 1% gentamycin. Epidermal melanocytes (MC) were isolated at the Department of Dermatology and Allergology, University of Szeged, Hungary, as described before [54,55]. Briefly, breast or trunk skins of healthy individuals undergoing plastic surgery were used after informed consent. The epidermis was denuded from the dermis after digestion for 48 h at 4 °C in grade II dispase solution (Roche Diagnostics, Mannheim, Germany) and dissociated using 0.25% trypsin at 37 °C for 20 min. After filtering through a 100-μm nylon mesh (BioDesign, Saco, ME, USA) and centrifuged at 200× *g* for 10 min at 4 °C, the cells were harvested to 2 × 10^6^ cell/mL density, and then digested with 0.01% trypsin-0.05% EDTA (ethylenediaminetetraacetic acid) to release melanocytes. Finally, they were grown in Mel-mix medium containing equal portions of serum-free lymphocyte medium (AIM-V) and keratinocyte serum-free medium supplemented with 2.5 ng/mL epidermal growth factor (EGF), 25 μg/mL bovine pituitary extract (BPE), 1% L-glutamine, 1% gentamycin and 2.5% fetal bovine serum (FBS). Melanocyte origin of isolated cells was proved by tyrosinase-related protein-1 expression. All cultures were incubated at 37 °C in humidified atmosphere containing 5% CO_2_. Media were changed every 2–3 days, and cells were harvested for passage or examinations at ~90% confluence. All reagents were from Life Technologies (Thermo-Fisher Scientific, TS Labor, Budapest, Hungary), except where otherwise indicated.

### 4.3. RNA Isolation, cDNA Transcription and Real-Time PCR

RNAs were isolated using the RNeasy Mini Kit (Qiagen, Venlo, Netherlands). Cells grown in 75 cm^2^ flasks at ~90% confluence were washed with 0.05 M phosphate-buffered saline (PBS) and released using trypsin-EDTA as above, collected in 5 mL of their relevant culture media, centrifuged at 300× *g* for 10 min, suspended in PBS and centrifuged again. The cells stored on ice in PBS were lysed by homogenization in mixture of 600 µL RLT buffer and 600 µL 70% ethanol. Lysates were filled into the columns and centrifuged with 8000× *g* for 30 s followed by adding 350 µL RW1 washing buffer. Then a mixture of 70 µL RDD and 10 µL DNase stock solution was added and the samples were incubated at 24 °C for 20 min followed by mixing with 350 µL of RW1 buffer. Spinning down again and adding to the columns with 500 µL RPE buffer the samples were collected into new tubes and centrifuged, then the latter step was repeated again until the membrane dried out. Finally, columns were put in new Eppendorf tubes containing 50 µL RNase free water, incubated at 24 °C for 2 min and then centrifuged again for 1 min. Total RNA concentration was measured at 260 nm using NanoDrop 1000 Spectrophotometer (Thermo-Fisher).

For reverse transcription (RT), 1 µg total RNA sample was mixed with 2 µL 10× RT buffer, 2 µL random primer solution and 0.5 µL dNTP solution completed with ultra-pure water to 19 µL. Samples were denatured at 70 °C for 5 min then cooled to 4 °C in the PCR instrument (Thermo-Fisher, Applied Biosystems, Waltham, MA, USA) and complemented with 1 µL reverse transcriptase polymerase solution. For cDNA synthesis, samples were heated to 42 °C for 1 h than to 85 °C for 10 min, finally cooled down to, and stored at, 4 °C. For negative controls either no enzyme or water substitution of RNA were used.

For qRT-PCR, TaqMan probes including relevant primers, listed in Table 4, were used to quantify connexin gene expression in relation to that of ACTB (encoding β-actin) housekeeping gene. These reagents were obtained from IDT (Integrated DNA Technologies, Skokie, IL, USA). All samples were tested in at least 3 parallels using Eppendorf PCR plates containing 5.25 µL ultra-pure water, 7.5 µL PCR Master Mix, 0.75 µL primer probe solution and 1.5 µL cDNA. Plates were covered, centrifuged and PCR was done for 40 cycles.

### 4.4. Melanocytic Tissue Lesions and Tissue Microarray

Tissue samples routinely fixed in 4% formaldehyde and then embedded in paraffin wax representing cases of 16 common nevi, 58 dysplastic nevi, 49 primary melanomas (22 “thin”, ≤1mm including 3 in situ melanomas and 27 “thick”, >1 mm vertical thickness) and 19 melanoma metastases diagnosed between 2003 and 2006 at the Department of Dermatology and Allergology, University of Szeged, Hungary, were tested [56]. In critical cases, diagnoses were based on consensus between at least two expert dermatopathologists (Erika Varga and Istvan Balazs Nemeth). The mean age of patients was 33.8 (11–64) years for common nevi (6 males and 10 females), 34.3 (12–76) years for dysplastic nevi (33 males and 25 females), 63.7 (25–91) years for primary melanomas (23 males and 26 females) and 65.6 (43–87) years for metastatic melanomas (4 males and 15 females). Clark levels sorted melanomas into level I: 6, II: 13, III: 20, IV: 7 and V: 3 cases. For thin melanomas, the mean thickness was 0.530 mm (0.157–0.988 mm) and the mean mitotic index was 2.9 (0–11). For thick melanomas, the mean tumor thickness was 4.835 mm (1.064–15.806 mm) and the mean mitotic index 14.9 (2–45). Patients’ data were coded and handled in accordance to the ethical regulations of the Institutional Review Boards at the Department of Dermatology and Allergology, University of Szeged and of Semmelweis University, Budapest (approval number: KL-37/2006).

Tissue cores of 2 mm diameter were collected into 5 pieces of 70-sample tissue microarray (TMA) blocks from representative areas based on hematoxylin and eosin (H&E) stained slides using the computer driven TMA Master (3DHISTECH Kft., Budapest, Hungary) [57,58]. Small lesions were included in single tissue cores. From sizable melanomas, duplicate or triplicate cores were taken by systematically selecting samples from the vertical tumor front, the mid-region and the uppermost region closest to or including the epidermis. Case numbers listed above were used for final evaluation after leaving out damaged or non-representative samples.

### 4.5. Immunocyto- and Histochemistry

For immunocytochemistry, cultured cells were washed in 0.05 M Tris-buffered saline (TBS) pH7.4 then released from adhesion by using 0.01% trypsin-0.02% EDTA, passed into 8-well culture slides (Falcon, Becton, Dickinson and Company, Franklin Lakes, NJ, USA) and grown until ~75% confluence. Cultures were then washed in TBS, fixed for 10 min at −20 °C in cold methanol, dried and washed again for 10 min in TBS before immunostaining. Parallel samples fixed in TBS buffered 4% formaldehyde for 10 min were also tested, after permeabilization in TBS containing 0.2% Triton-X-100 (TBST) for 10 min. Nonspecific binding sites were blocked using 5% bovine serum albumin (BSA) in TBS for 20 min and cultures were incubated overnight using the primary antibodies listed in Table 5.

For immunohistochemistry 4 µm thick sections cut from TMA blocks mounted on adhesive glass slides (Thermo-Fisher) were routinely dewaxed in xylene and ethanol series and treated for endogenous peroxidase blocking in a 0.5% hydrogen peroxide in methanol for 20 min. For antigen retrieval, the slides were heated in 0.01M Tris-0.1M EDTA buffer pH 9.0 (TE) at ~100 °C for 50 min using a JT 366 microwave oven (Whirlpool, Benton Harbor, MI, USA). Occasionally, short digestion followed in 0.005% trypsin-phenol red solution (Thermo-Fisher, Gibco) for 10–20 s. Nonspecific binding sites were saturated using 5% BSA-TBST solution for 30 min, followed by the incubation with the primary antibodies overnight (Table 5). Connexin isotype specific antibodies were selected based on the in silico mRNA expression data and our preliminary immunostaining tests results with preference of antibodies supported by Western blot data. Antibodies which were not found to be specific or reliable based testing in Western blots and/or in human archived tissues are listed in Table S1. For immunoperoxidase staining, the Novolink polymer-peroxidase complex (Leica-Novocastra, Newcastle Upon Tyne, UK) method was used including an incubation with the post-primary blocking reagent then with the polymer for 30 min each. Enzyme activity was visualized using a hydrogen peroxide/AEC (3-amino-9-ethylcarbazole, red) substrate-chromogen solution at pH 4.5 under microscopic control for 3–5 min. This permitted us to separate brown melanin pigments (the same color as 3,3’-diaminobenzidine - DAB chromogen would be) from the specific immunoreaction. Finally, the slides were counterstained using hematoxylin and finally coverslip-mounted using Faramount aqueous medium (Dako, Glostrup, Denmark).

For immunofluorescence, rabbit primary antibodies were mixed with mouse anti-vimentin (clone: V9; in 1:200), Ki67 (Mib1, 1:200) or Melan A (1:200) for highlighting cell structure, proliferating cells or confirming melanocytic cell origin, respectively. Primary antibodies were detected by combining Alexa Fluor 488/or 546 conjugated goat anti-mouse/or -rabbit (IgG H + L) secondary antibodies for 90 min diluted in TBST followed by nuclear DNA staining using Hoechst (Bisbenzimide, 1:500; Sigma-Aldrich, St. Louis, MO, USA). Between incubations, tissue sections or cell cultures were washed 3 × 2 min in TBST. Finally, samples were coverslip-mounted using fluorescent mounting medium (Dako).

Immunoreactions using some other connexin isotype specific antibodies, indicated by mRNA expression studies including Cx31(*GJB3*), Cx31.1(*GJB5*), Cx45(*GJC1*) and Cx31.9(*GJD3*), were negative.

### 4.6. Scoring of Immunoreactions in Tissue Sections

Connexin immunoreactions in tissue sections were scored based on the proportion of tumor cells showing obvious immunostaining. The individual scoring thresholds used were: (A) for Cx43 and Cx30.2, score 0 (negative): ≤5%; score 1: 5–25%; score 2: >25%; (B) for Cx46 and Cx26, score 0: ≤10%; score 1: 10–50%; score 2: 50–75%; score 3: >75%. In Cx32 and Cx30 stained samples besides tumor cells their microenvironments were also scored as 0: ≤5% (negative) and 1: >5% (positive). In epidermal keratinocytes, positive cell layers were assessed according to Haass et al. (2010) [15]: score 0: negative; score 1: stratum granulosum (SG); score 2: uppermost layers (UL); score 3: suprabasal layers (SL); score 4: all layers (AL).

### 4.7. Dye Transfer Analysis

Free-floating cells were double fluorescence labelled using a permanent membrane phospholipid marker red dye Dil (excitation/emission peaks: 549/565 nm) and the membrane permeable calcein acetoxymethyl ester (calcein AM), which is converted into hydrophilic green dye calcein (Mw: 465 kDa; excitation/emission peaks: 495/515 nm) by intracellular esterases, enabling it to pass through gap junction channels between adjacent cells.

From each sample 2 × 10^6^ cells, suspended in 1 mL PBS, were mixed either with 0.5 µM calcein AM and 9 µM Dil or only single labelled with calcein, and cultured at 37 °C for 30 min as described above to allow the formation of intracellular hydrophilic calcein [59]. After 3× repeated washing in PBS and centrifugation, 10^6^ double stained, single (calcein) stained and unlabeled cells were collected in 300 µL PBS, respectively for controls. Then, 10^5^ double stained cells were mixed with 9 × 10^5^ unstained cells and grown at 37 °C for 5 h in 25 cm^2^ cell culture flasks until reaching confluency. After washing in PBS, 0.01% trypsin-0.02% EDTA treatment, repeated washing and centrifugation 3× mixed cells were resuspended in 300 µL PBS for flow cytometry. Di1 positive cell fraction represented the originally double-stained cells, while the proportion of single calcein labelled cells indicated the intensity of direct cell-cell communication through gap junction channels.

### 4.8. Statistics and Hierarchical Cluster Analysis

Analysis of in silico gene expression and qRT-PCR was done using the unpaired *t*-test of the SPSS software package (IBM, Armonk, NY, USA). Immunohistochemistry scores were assessed after dichotomization using the two-sided Fisher’s exact test by pairwise comparison of neighboring categories: e.g., nevi vs. melanomas and along the common nevus–dysplastic nevus–thin melanoma (≤1 mm)–thick melanoma (>1 mm) and metastatic melanoma progression sequence. Possible correlations among connexin expression profiles and tumor progression groups were also tested using unweighted hierarchical cluster analysis within the “R” statistical environment (R 15.2.0; R Foundation, Vienna, Austria).

## 5. Conclusions

The differential and diverse Cx expression revealed in this study is likely to result from the known heterogeneity of melanocytic tumors. The most significant feature of melanoma evolution is the lost Cx43 expression at malignant transformation and its moderate recurrence in metastatic melanomas, which mostly define the varying metabolic communication. Cx expression-based hierarchical clustering supports dysplastic nevi as risk factors of malignant transformation by some being sorted into the same group as thin melanomas. Elevation of diffuse cytoplasmic Cx26 and paranuclear or cell membrane-bond Cx32 and paranuclear Cx30.2 during melanoma progression, the induced Cx43, Cx26 and Cx30 proteins in melanoma adjacent epidermis, and Cx43 in the tumor flanking endothelia, may reflect bidirectional adaptive interactions between the tumors and their microenvironment. Further studies should focus on correlating Cx expression and functions with the epi-/genetic background of melanocytic tumors.

## Figures and Tables

**Table 1:** Connexin mRNA expression data of relevant melanocytic tissue lesions found in GEO (Gene Expression Omnibus) database.

GEO Code	Platform	Materials	Reference
GSE3189	Affymetrix Human Genome U133A	clinical; 18 nevi, 45 melanomas	Talantov et al. 2005 [26]
GSE7553	Affymetrix Human Genome U133 Plus 2.0	clinical; 2 *in situ*, 14 primary, 40 metastatic melanomas	Riker et al. 2008 [27]
GSE8401	Affymetrix Human Genome U133A	mouse xenograft model; 31 primary, 52 metastatic melanomas	Xu et al. 2008 [28]

**Table 2:** Number of probes for connexin genes in Affymetrix Human Genome arrays examined.

Connexin Gene	Connexin Protein	Affymetrix Human Genome U133A	Affymetrix Human Genome U133 Plus 2.0
*GJA1*	Cx43	1	1
*GJA3*	Cx46	1	0
*GJA4*	Cx37	2	3
*GJA5*	Cx40	1	2
*GJA8*	Cx50	1	1
*GJA9*	Cx59	1	1
*GJA10*	Cx62	0	1
*GJB1*	Cx32	1	1
*GJB2*	Cx26	0	1
*GJB3*	Cx31	3	3
*GJB4*	Cx30.3	1	1
*GJB5*	Cx31.1	1	2
*GJB6*	Cx30	0	1
*GJC1*	Cx45	1	5
*GJC2*	Cx47	2	2
*GJD2*	Cx36	1	1
*GJD3*	Cx31.9	0	2
*GJD4*	Cx40.1	0	1

**Table 3:** Summary of in silico analysis, as well as mRNA and protein expression results of cell culture and tissue samples.

Connexin Isotype		In Silico Analysis of mRNA Expression			qRT-PCR (dCt)/Immunocytochemistry				Immunohistochemistry (% positive)
Cx Gene	Cx Protein	GSE3189 (Clinical)	GSE7553 (Clinical)	GSE8401 (Xenograft)	MC	WM983/A (vertical)	HT199 A2058 (metastatic)		Nevus vs. Melanoma
*GJA1*	Cx43	N > M	PM > MMet	PM > MMet	3.34/CP, PN, CM	no/no	no/no	no/no	CM: 75 > 5
*GJA3*	Cx46	low			11.75/CP	8.56/CP	6.99/PN	9.85/CP	PN: 100 vs. 96
*GJB1*	Cx32	N < M	PM ~ MMet	PM ~ MMet	no/no	* 5.65/CP	** 5.25/CP	** 4.92/CP	CP: 88 vs. 97 CM: 17 vs. 11 PN: 0 < 15
*GJB2*	Cx26		PM > MMet		4.53/CP	3.78/no	8.51/CP/CM	12.45/CP	CP: 65 < 85 DF: 37 < 66 PN: 63 > 34
*GJB3*	Cx31	N > M	PM > MMet	PM > MMet	no	3.23	11.64	no	Not tested
*GJC1*	Cx45	N ~ M	PM ~ MMet	low	8.16	no	7.65	8.31	Not tested
*GJC3*	Cx30.2		Not included		12.38/PN	12.93/no	11.29/PN	12.41/no	PN: 3 < 23

> or <, significant difference; ~, no significant difference; N, nevi; M, melanomas; PM, primary melanomas; MMet, metastatic melanomas; MC, primary melanocyte culture; CP, cytoplasmic immunoreaction; PN, paranuclear; CM, cell membrane. * Positive with all three *GJB1* TaqMan probes; ** negative with psHs.PT.56a.4848609 probe, but positive with Hs00939759s1 and Hs04259568s1 probes.

**Table 4:** Predesigned primers used for qRT-PCR testing of connexin target exons.

Gene	Primer
ACTB exon 1-2	Hs.PT.56a.22214847
*GJA1* exon 1-2	Hs.PT.56a.38338544
*GJA3* exon 1-2	Hs.PT.56a.28039214
*GJA4* exon 1-2	Hs.PT.56a.987509
*GJB1* exon 1-3	Hs.PT.56a.4848609
*GJB1* exon 2-2	Hs00939759_s1
*GJB1* exon 2-2	Hs04259568_s1
*GJB2* exon 2-2	Hs.PT.56a.505396.g
*GJB3* exon 2-2	Hs.PT.56a.39590773.g
*GJB4* exon 1-2	Hs.PT.56a.26779601
*GJB5* exon 1-2	Hs.PT.56a.321621
*GJB6* exon 5-6	Hs.PT.56a.4153325.g
*GJB7* exon 1-2	Hs.PT.56a.39266451
*GJC1* exon 3-4	Hs.PT.56a.19845193
*GJC2* exon 1-2	Hs.PT.56a.18725226
*GJC3* exon 2-2	Hs.PT.56a.26377588.g
*GJD2* exon 1-2	Hs.PT.56a.39459544
*GJD3* exon 1-1	Hs.PT.56a.24558172.g
*GJD4* exon 1-2	Hs.PT.56a.40171761

**Table 5:** Connexin isotype specific polyclonal rabbit primary antibodies used for immunocyto-/histochemistry.

Connexin Gene	Connexin Protein	Antibody	Dilution
*GJA1*	Cx43	Connexin 43 Cell Signaling #3512	1:100
*GJA3*	Cx46	Anti-GJA3 (N-term) Sigma-Aldrich SAB1300557	1:75
*GJB1*	Cx32	Anti-GJB1 Sigma-Aldrich HPA010663	1:30
*GJB2*	Cx26	Anti-Connexin 26 Millipore AB8143	1:1000
*GJB6*	Cx30	Anti-GJB6 Sigma-Aldrich HPA014846	1:75
*GJC3*	Cx30.2	Anti-GJE1 Sigma-Aldrich AV36638	1:500

## Supplementary Materials

The following are available online at http://www.mdpi.com/2072-6694/11/2/165/s1, Table S1: Antibodies tested in human skin tissues but were not found to be specific or reliable in this study.
