# Supplementary material for: Exploring Differential Connexin Expression across Melanocytic Tumor Progression Involving the Tumor Microenvironment
Source: Cancers (Basel). 2019 Feb 1;11(2):165. doi: 10.3390/cancers11020165 (PMC6406766; doi:10.3390/cancers11020165)
Supplement: Supplementary file 1 [file cancers-11-00165-s001.pdf]

# Supplementary material: Exploring Differential Connexin Expression Across Melanocytic Tumor Progression Involving the Tumor Microenvironment

**Gergo Kiszner, Peter Balla, Barna Wichmann, Gabor Barna, Kornelia Baghy, Istvan Balazs Nemeth, Erika Varga, Istvan Furi, Bela Toth and Tibor Krenacs**

**Table S1.** Antibodies tested in human skin tissues but were not found to be specific or reliable in this study.

| Connexin Gene | Connexin Protein | Antibody | Dilution |
|---|---|---|---|
| GJA1 | Cx43 | Anti-Cx43, mouse clone CXN-6<br>Santa Cruz sc-59949 | 1:300 |
| GJA5 | Cx40 | Anti-40 (H-116) rabbit polyclonal<br>Santa Cruz sc-28658 | 1:75 |
| GJB3 | Cx31 | Anti-GJB3, mouse clone 3B4-1B3<br>Sigma-Aldrich WH0002707M1 | 1:350 |
| GJB4 | Cx30.3 | Anti-GJB4, mouse clone 1E3-1C12<br>Sigma-Aldrich WH0127534M1 | 1:1800 |
| GJB7 | Cx25 | Anti-GJB7 (96-110) rabbit polyclonal<br>Sigma-Aldrich SAB1103736 | 1:1000 |
| GJC2 | Cx47 | Anti-GJC2 rabbit polyclonal<br>Sigma-Aldrich SAB2100924 | 1:200 |
| GJC3 | Cx30.2 | Anti-GJC3 rabbit polyclonal<br>Sigma-Aldrich HPA015024 | 1:50 |
| GJD2 | Cx36 | Anti-GJD2 rabbit polyclonal<br>Sigma-Aldrich HPA014355 | 1:50 |
| GJD3 | Cx31.9 | Anti-GJD3 rabbit polyclonal<br>Sigma-Aldrich HPA043159 | 1:200 |

© 2019 by the authors. Licensee MDPI, Basel, Switzerland. This article is an open access article distributed under the terms and conditions of the Creative Commons Attribution (CC BY) license (http://creativecommons.org/licenses/by/4.0/).
